# Socioeconomic Factors Influencing Hospitalized Patients with Pneumonia Due to Influenza A(H1N1)pdm09 in Mexico

**DOI:** 10.1371/journal.pone.0040529

**Published:** 2012-07-11

**Authors:** Toshie Manabe, Anjarath Lorena Higuera Iglesias, Maria Eugenia Vazquez Manriquez, Eduarda Leticia Martinez Valadez, Leticia Alfaro Ramos, Shinyu Izumi, Jin Takasaki, Koichiro Kudo

**Affiliations:** 1 Disease Control and Prevention Center, National Center for Global Health and Medicine, Tokyo, Japan; 2 Department of Clinical Epidemiology Investigation, Research Institute, National Institute of Respiratory Diseases, Mexico City, Mexico; 3 Department of Pathology, National Institute of Respiratory Diseases, Mexico City, Mexico; 4 Department of Epidemiology, National Institute of Respiratory Diseases, Mexico City, Mexico; University of Liverpool, United Kingdom

## Abstract

**Background:**

In addition to clinical aspects and pathogen characteristics, people's health-related behavior and socioeconomic conditions can affect the occurrence and severity of diseases including influenza A(H1N1)pdm09.

**Methodology and Principal Findings:**

A face-to-face interview survey was conducted in a hospital in Mexico City at the time of follow-up consultation for hospitalized patients with pneumonia due to influenza virus infection. In all, 302 subjects were enrolled and divided into two groups based on the period of hospitalization. Among them, 211 tested positive for influenza A(H1N1)pdm09 virus by real-time reverse-transcriptase-polymerase-chain-reaction during the pandemic period (Group-pdm) and 91 tested positive for influenza A virus in the post-pandemic period (Group-post). All subjects were treated with oseltamivir. Data on the demographic characteristics, socioeconomic status, living environment, and information relating to A(H1N1)pdm09, and related clinical data were compared between subjects in Group-pdm and those in Group-post. The ability of household income to pay for utilities, food, and health care services as well as housing quality in terms of construction materials and number of rooms revealed a significant difference: Group-post had lower socioeconomic status than Group-pdm. Group-post had lower availability of information regarding H1N1 influenza than Group-pdm. These results indicate that subjects in Group-post had difficulty receiving necessary information relating to influenza and were more likely to be impoverished than those in Group-pdm. Possible factors influencing time to seeking health care were number of household rooms, having received information on the necessity of quick access to health care, and house construction materials.

**Conclusions:**

Health-care-seeking behavior, poverty level, and the distribution of information affect the occurrence and severity of pneumonia due to H1N1 virus from a socioeconomic point of view. These socioeconomic factors may explain the different patterns of morbidity and mortality for H1N1 influenza observed among different countries and regions.

## Introduction

In March 2009, Mexico was the first country to raise the international alert about the outbreak of influenza A(H1N1)pdm09 virus infection [1]. The pandemic spread rapidly worldwide; however, the number of severe and fatal cases differed among different countries and regions [2]. In Mexico, there were large numbers of hospitalized patients with acute and severe illness, and fatalities occurred [1], [2], [3], [4], [5]. Many patients with influenza-like symptoms, including pneumonia, presented to the National Institute of Respiratory Disease (INER), Mexico City, Mexico; a number of these required hospitalization owing to the severity of the illness, and there were fatalities, especially at the early stage of the pandemic [1]. After the announcement of the post-pandemic period for influenza A(H1N1)pdm09 by the World Health Organization (WHO) [6], effective management of the influenza pandemic continued to be a major concern. Clinical preparedness for A(H1N1)pdm09 was based on an understanding of the pathogenic characteristics of the virus and host immune-response patterns as well as the ability to undertake clinical interventions and ordinary individuals' knowledge about prevention; all of these factors were thought to have been at a developed stage in Mexico following the experiences of the 2009 influenza pandemic. However, a similar number of hospitalized patients with influenza-associated pneumonia presented again to the INER in the post-pandemic period [7].

Various factors affecting occurrence of pneumonia, disease severity, and mortality associated with A(H1N1)pdm09H1N1 have been reported from a clinical viewpoint [8], [9], [10], [11]. Other underlying conditions, including such environmental and socioeconomic factors as education and poverty, are also thought to affect the disease morbidity and mortality [12], [13]. However, only limited data are available regarding the influence of socioeconomic factors on the occurrence of pneumonia related to influenza virus infection. Previously, we reported that the number of days between symptom onset and oseltamivir treatment affects the occurrence and severity of pneumonia due to H1N1 influenza [14]. Delayed treatment is associated with socioeconomic difficulties of INER patients [14]. A study in Canada also showed that delayed antiviral treatment is independently associated with disease severity [15]. We hypothesize that some risk factors affecting the continued occurrence of pneumonia in the post-pandemic period in Mexico, including delay in seeking healthcare, need to be addressed from the socioeconomic rather than clinical point of view. These factors may explain the different mortality and morbidity patterns for A(H1N1)pdm09 in different countries and regions.

The aim of the present study was to assess how socioeconomic and living conditions relate to the disease severity of H1N1 influenza, including pneumonia, in Mexico.

## Materials and Methods

### Study design

The survey was conducted at the INER, Mexico City between December 2010 and April 2011, and it included follow-up consultation with subjects or their relatives using structured questionnaires administered by physicians or trained medical staff. The subjects were former patients hospitalized in the INER for pneumonia due to A(H1N1)pdm09 who tested positive by real-time reverse-transcriptase-polymerase-chain-reaction (RT-PCR) during the pandemic period; patients hospitalized for pneumonia who tested positive for influenza A virus during the post-pandemic period served as a comparison group. Patients with pneumonia caused by primary bacterial infection were excluded. All subjects were treated with oseltamivir. The pandemic period was defined as April 2009 to July 2010, and the post-pandemic period was defined as August 2010 to March 2011, in accordance with the declaration of the post-pandemic period by the WHO [9]. No subjects were hospitalized in both the pandemic and post-pandemic periods.

The questionnaire was designed to collect data on the demographic characteristics of subjects, socioeconomic status, living environment, and information relating to A(H1N1)pdm09, as well as related clinical data. Socioeconomic status was classified in terms of daily income while living environment was defined in terms of numerous factors associated to living conditions, including area in which subjects lived (i.e., location), house size and construction material, among other factors. All questions were either closed-ended or multiple choice. Each variable was compared between subjects hospitalized in the pandemic period (Group-pdm) and those hospitalized in the post-pandemic period (Group-post). In addition, factors affecting access to health care were evaluated. Socioeconomic level was classified based on daily income and on the ability of household income to pay for utilities, food, and medical services according to the Social Gap Index by the CONEVAL (Consejo Nacional de Evaluación de la Política de Desarrollo Social) [16]. Location was defined by the accessibility to public service which was also evaluated according to the Social Gap Index.

All subjects provided written informed consent. Ethical approval was provided by the Institutional Review Board of the National Institute of Respiratory Diseases, Mexico City and the National Center for Global Health and Medicine, Tokyo. The investigators maintained the datasets in password-protected systems and have preserved the anonymity of the subjects when presenting data.

### Statistical Analysis

Data from the surveys were double-entered and analyzed using SPSS ver. 19 (IBM, Armonk, NY, USA). For categorical variables, frequencies were compared using the chi-square test and Fisher's exact test. For determination of independent factors for the time to seeking healthcare, multivariate regression analysis was conducted using a stepwise selection method included all variables in baseline characteristics, socioeconomic status, living environment, and information relating to A(H1N1)pdm09, and related clinical data, if p <0.1 in univariate analysis. For all analyses, significance levels were two-tailed, and p value of <0.05 was considered significant.

## Results

### General and health-related backgrounds for study subjects

In all, 302 subjects who were hospitalized with pneumonia between April 2009 and March 2011 and received follow-up consultation during the study period agreed to participate in the present survey. Among them, 211 (69.9%) were hospitalized during the pandemic period (Group-pdm) and 91 (30.3%) in the post-pandemic period (Group-post). The general backgrounds of subjects are listed (Table 1). The median ages of subjects in Group-pdm and Group-post were 38.5 (range, 0–90) years and 42.0 (range, 2–91) years, respectively. There was a higher percentage of younger subjects in Group-pdm than in Group-post (p = 0.001). There was no significant sex difference between the groups (p = 0.354). Approximately 17% of the subjects had received no education, and there was no significant difference in education level between the groups (p = 0.356). Although unemployment was higher in Group-post (18.7%) than in Group-pdm (8.6%), the occupations were not significantly different between the groups (p = 0.437). The socioeconomic level of 70.5% of all subjects was low, whereas 29.5% were of middle socioeconomic level (p = 0.332).

**Table 1:** General and health-related backgrounds of study subjects.

	Pandemic period*	Post-pandemic period*	Total	p value
	N = 211 (69.9%)	N = 91 (30.3%)	N = 302 (100.0%)	
**Gender**				
Male-no. (% of group)	128 (60.7)	50 (54.9%)	178 (58.9%)	0.354
**Age – median (range)**	38.5 (0–90)	42.0 (2–91)	39.0 (0–91)	0.003
**Age group – no. (% of group)**				0.001
<18	45 (21.4)	4 (4.4)	49 (16.3)	
18-<50	108 (51.2)	52 (57.1)	160 (53.0)	
50-<65	43 (20.4)	22 (24.2)	65 (21.5)	
≥65	15 (7.1)	13 (14.3)	28 (9.3)	
**Education background – no. (% of group)**				0.356
None	34 (16.4)	17 (17.9)	51 (16.9)	
Primary school	57 (27.5)	25 (26.3)	82 (27.2)	
Secondary school	49 (23.7)	16 (16.8)	65 (21.5)	
High school	33 (15.9)	19 (20.0)	52 (17.2)	
University	30 (14.5)	12 (12.6)	42 (13.9)	
Graduate school	2 (1.0)	3 (3.2)	5 (1.7)	
Technical school	2 (1.0)	3 (3.2)	5 (1.7)	
**Occupation – no. (% of group)**				0.437
Unemployed	18 (8.6)	17 (18.7)	35 (11.7)	
Retired	1 (0.5)	2 (2.2)	3 (1.0)	
Student	28 (13.4)	3 (3.3)	31 (10.3)	
Housewife	53 (25.4)	30 (33.0)	83 (27.7)	
Governmental employee	4 (1.9)	3 (3.3)	7 (2.3)	
Employee by private company	30 (14.4)	15 (16.5)	45 (15.0)	
Commercial	33 (15.8)	5 (5.5)	38 (12.7)	
Self-employed (small business)	28 (13.4)	3 (3.3)	31 (10.3)	
**Socioeconomic level** †				0.332
Low	144 (72.4)	69 (67.0)	213 (70.5)	
Middle	55 (27.6)	34 (33.0)	89 (29.5)	
High	0 (0.0)	0 (0.0)	0 (0.0)	
**Heath-related background**				
**Vaccination (seasonal influenza)**				0.203
Vaccinated in 2009	4 (1.9)	6 (6.6)	10 (3.3)	
Vaccinated in 2010	42 (19.9)	20 (22.0)	62 (20.5)	
Vaccinated in 2011	8 (3.8)	3 (3.5)	11 (3.6)	
**Smoking**				0.002
smoker	18 (8.5)	21 (23.1)	39 (12.9)	
ex-smoker	19 (9.0)	8 (8.8)	27 (8.9)	
**Chronic respiratory illness on medication**	6 (2.8)	40 (44.0)	46 (15.2)	0.000
**Days from symptom onset to initiation of treatment – median (range)** ‡	6.0 (0–35)	5.0 (0–29)	6.0 (0–35)	0.379

* Pandemic period, between April 2009 and July 2010; Post-pandemic period, between August 2010 and the end of survey period.

† Socioeconomic level evaluated by ability to pay for utilities, food, and medical service: low income, cannot cover electricity, water, telephone, house rent, foods, any medical service; middle income, can cover a part of electricity, water, telephone, house rent, and foods, but not any medical service; high income, can cover utility and adequate goods, and medical services.

‡ Number of days from symptom onset to initiation of oseltamivir administration.

The health-related background details of subjects are presented in Table 1. The rate of seasonal influenza vaccination was approximately 20% in both groups in 2010; however, the vaccinated populations in 2009 and 2011 were smaller than in 2010. There were significantly more smokers in Group-post (23.1%) than in Group-pdm (8.1%) (p = 0.002). Medication for chronic respiratory illness was being taken by 2.8% of Group-pdm subjects and 44.0% of Group-post subjects (p<0.001). The median number of days to initiation of oseltamivir administration from symptom onset for all subjects was 6 days (range, 0–35) and there was no significant difference between the groups (p = 0.379).

### Economic factors

The detailed economic situation of the subjects was defined by the Social Gap Index of COVEVAL (Table 2). The ability of household income to pay for all utility services (light, gas, water, sewerage, and telephone) or some (two or three of the five) was significantly lower in Group-post than in Group-pdm (p<0.001). The ability of household income to pay for all foods (meat, egg, milk, cereals, vegetables) or some foods (two or three of the five) was 59.7% and 40.3%, respectively, in Group-pdm vs. 27.8% and 56.7%, respectively, in Group-post (p<0.001); 15.6% of the subjects in Group-post stated that could not pay for any food from their income. The ability of household income to pay for all health care services (consultation, hospitalization, and medication) or some of them (consultation and hospitalization) were 28.8% and 67.6%, respectively, in Group-pdm vs. 17.8% and 38.9%, respectively, in Group-post (p<0.001); 82.8% of subjects were uninsured.

**Table 2:** Detailed economic status of study subjects.

	Pandemic period*i	Post-pandemic period*	Total	p value
	N = 211 (69.9%)	N = 91 (30.3%)	N = 302 (100.0%)	
**Income ability to pay for utility services** †				0.000
All services	76 (36.0)	16 (17.6)	92 (30.5)	
2–3 of all	133 (63.0)	41 (45.1)	174 (57.6)	
None	2 (0.9)	34 (37.4)	36 (11.9)	
**Income ability to pay for food** ‡				0.000
All necessaries	126 (59.7)	25 (27.8)	151 (50.2)	
2–3 of all	85 (40.3)	51 (56.7)	136 (45.2)	
None	0 (0.0)	14 (15.6)	14 (4.7)	
**Income ability to pay for Health care service**				0.000
Consultation, hospitalization, medication	60 (28.6)	16 (17.8)	76 (25.3)	
Consultation, hospitalization	142 (67.6)	35 (38.9)	177 (59.0)	
None	8 (3.8)	39 (43.3)	47 (15.7)	
**Health insurance**				0.144
None	179 (84.8)	71 (78.0)	250 (82.8)	
Government insurance§	17 (8.1)	8 (8.8)	25 (8.3)	
Private insurance	1 (0.5)	0 (0.0)	1 (0.3)	
Others	14 (6.6)	12 (13.2)	26 (8.6)	

* Pandemic period, between April 2009 and July 2010; Post-pandemic period, between August 2010 and the end of survey period.

† Income can pay for expense of utilities; light, gas, water, sewerage, telephone.

‡ Income can pay for expense of food; meat, egg, milk, cereals, vegetable.

§ Governmental insurance included workers in private organizations.

### Life environment

Most of the subjects in Group-pdm and Group-post lived in an area that provided all public services (p = 0.144), and there was no difference between the groups. In terms of housing status (Table 3), 39.8% and 30.8% of subjects in Group-pdm and Group-post lived for free in borrowed accommodation, whereas 18.5% and 34.1%, respectively, lived in rental accommodation; there was a significant difference in housing status between the groups (p = 0.011). Although most Group-pdm subjects lived in concrete houses or a combination of concrete and tinplate houses, more subjects in Group-post lived only in tinplate houses, and there was a significant difference in housing quality between the groups (p<0.001). The number of rooms and individuals living in a house were significantly different between the groups (p = 0.003 and p<0.001, respectively). The median number of individuals per room in each household was 2.5 (range, 0.29–8.0) in Group-pdm and 3.0 (range, 0.67–9.0) in Group-post, and there was a significant difference between the groups (p<0.001).

**Table 3:** Life environmental qualities of study subjects.

	Pandemic period*	Post-pandemic period*	Total	p value
	N = 211 (69.9%)	N = 91 (30.3%)	N = 302 (100.0%)	
**Location** †				0.144
All public services	187 (88.6)	75 (82.4)	262 (86.8)	
Partial public services	24 (11.4)	16 (17.6)	40 (13.2)	
**Housing**				0.011
Borrow without any payment	84 (39.8)	28 (30.8)	112 (37.1)	
Rent	39 (18.5)	31 (34.1)	70 (23.2)	
Pay for credit	16 (7.6)	2 (2.2)	18 (6.0)	
Own	72 (34.1)	30 (33.0)	102 (33.8)	
**House construction material**				<0.001
Concrete	179 (84.8)	70 (76.9)	249 (82.5)	
Tinplate	3 (1.4)	12 (13.2)	15 (5.0)	
Concrete and tinplate	29 (13.7)	9 (9.9)	38 (12.6)	
**Number of rooms in a house**				0.003
≤2	85 (40.3)	64 (70.3)	149 (49.3)	
3–5	121 (57.3)	26 (28.6)	147 (48.7)	
≥6	5 (2.4)	1 (1.1)	6 (2.0)	
**Number of individuals in a house**				<0.001
≤2	13 (6.2)	24 (26.4)	37 (12.3)	
3–5	167 (79.5)	57 (62.6)	224 (74.4)	
≥6	30 (14.3)	10 (11.0)	40 (13.3)	
**Number of individuals per a room, mean (range)**	2.5 (0.29–8.0)	3.0 (0.67–9.0)	2.5 (0.29–9.0)	<0.001

* Pandemic period, between April 2009 and July 2010; Post-pandemic period, between August 2010 and the end of survey period.

† Location was defined by the accessibility of public service which is also followed by the Social Gap Index.

### Information relating to H1N1 virus infection

The most common source of information about influenza A(H1N1)pdm09 was television in both groups (p = 0.706) (Table 4). Although subjects in Group-pdm were more likely than those in Group-post to be informed through radio (55.5% vs. 25.3%, p<0.001), there was no significant difference in the use of other sources of information between the groups.

**Table 4:** Availability of information related to influenza A(H1N1)pdm09.

	Pandemic period*	Post-pandemic period*	Total	p value
	N = 211 (69.9%)	N = 91 (30.3%)	N = 302 (100.0%)	
**Information resources**				
Newspaper	34 (16.1)	13 (14.3)	47 (15.6)	0.688
Television	170 (80.6)	75 (82.4)	245 (81.1)	0.706
Radio	116 (55.5)	23 (25.3)	139 (46.3)	0.000
Internet	4 (1.9)	3 (3.3)	7 (2.3)	0.355
Family and friends	22 (10.4)	15 (16.5)	37 (12.3)	0.141
Healthcare workers in Hospital	23 (10.9)	3 (3.3)	26 (8.6)	0.031
No information	1 (0.5)	3 (3.3)	4 (1.3)	0.083
**Received clear information how to prevent influenza?**	163 (77.3)	27 (29.7)	190 (62.9)	0.000
**Received the information for the necessity of quick access to healthcare.**	199 (94.3)	55 (60.4)	254 (84.1)	0.000

* Pandemic period, between April 2009 and July 2010; Post-pandemic period, between August 2010 and the end of survey period.

More Group-pdm subjects than Group-post subjects received clear information about methods of prevention of influenza A(H1N1)pdm09 (77.3% vs. 29.7%, respectively, p<0.001) as well as information regarding the necessity for early access to health care (94.3% vs. 60.4%, respectively, p<0.001).

### Delay in seeking health care from symptom onset in hospitalized patients with pneumonia

In the multivariate regression analysis, the number of household rooms, information regarding the necessity for quick access to health care, and housing construction materials were independent factors that tended to be associated with the number of days from symptom onset to the initiation of antiviral treatment (Table 5). Since the INER administered antiviral treatment soon after hospital admission to hospitalized patients with pneumonia, the number of days to initiation of antiviral treatment was practically the same as the number of days from symptom onset to first access to formal health care.

**Table 5:** Factors relating to delayed seeking of health care, using multivariate analysis.

	Coefficient	Standard error	t value	p value	95% confidence interval
Constant	−10.246	4.351	−2.355	0.022	−18.985–−1.508
Number of rooms in house	3.798	0.895	4.242	0.000	2.000–5.597
Received information about necessity of quick access to health care during pandemic period	4.741	1.986	2.387	0.021	0.751–8.730
House construction material*	3.056	1.473	2.075	0.043	0.097–6.015

* House constructed of concrete, tinplate, and combination of concrete and tinplate.

## Discussion

Low awareness of the importance of early access to healthcare and difficulty separating oneself from other individuals in a household owing to poverty are possible reasons for hospitalized pneumonia due to influenza virus infection in the post pandemic period.

INER is a tertiary medical organization for the care of patients with respiratory illness, and it provides medical services mainly to uninsured individuals in the metropolitan area of Mexico City and neighboring states. Most patients who visit the INER have a similar low socioeconomic level, demographic characteristics, and educational background [17], including the subjects in the present study (Table 1). A detailed evaluation of socioeconomic similar subjects using the Social Gap Index of CONEVAL [16] revealed that <1% in Group-pdm were unable to pay for either utility services or food; there was a greater number of such subjects in Group-post (Table 2). Moreover, approximately half of the subjects in Group-post were unable to pay for health-care services. In contrast to Group-pdm subjects, those in Group-post lived in houses constructed of tinplate with fewer rooms, and there was also a greater number of individuals sharing the same house (Table 3). These results reflect the situation of many poor people in Mexico City, who live together with relatives and friends and help one another in their daily lives, including payments for utilities and food [19]. The subjects in Group-post were more likely to be impoverished than those in Group-pdm and showed a greater tendency to engage in mutual support. Economic difficulties and the inability to pay for treatment create problems in accessing formal health care in the early stage of any illness, even if the cause is acute viral infection; these factors can lead to the delayed initiation of appropriate treatment. For people without health-care insurance and who are paid on a daily basis, it is especially hard to stop work and seek medical assistance, even for a day. As a result, by the time they present to a hospital, their disease has progressed and may have become severe. This was the situation for patients in both Group-pdm and Group-post; however, those in Group-post faced greater poverty. There was a greater number of patients facing economic difficulties in Group-post than in Group-pdm, which indicates that subjects in the former group may have experienced more problems in accessing early health care. We previously showed that patients with severe pneumonia had a lower socioeconomic level and delayed initiation of oseltamivir treatment [14]. Patients in Group-post lived in houses with fewer rooms, but they also lived together with a greater number of other individuals (Table 3). This reflects not only the socioeconomic level of the subjects, but also an increased risk for human-to-human transmission of the influenza virus.

In Mexico, rural poverty is concentrated in southern areas of the country [19], [20]. Especially during the early stage of the influenza outbreak in 2009, there was a high rate of infection in populations in areas of rural poverty in the south including Mexico City [21]. However, Mexico City is not a single metropolitan area but a growing megalopolis. The city incorporates surrounding areas of poverty, and low- and middle-income communities live in close proximity in the same area. Most of the subjects in the present study were impoverished; however, >80% of them were located in areas with access to all public services, and there was no significant difference between the subjects in Group-pdm and Group-post (Table 3). This is typical of the unique living environment in Mexico City, and it reflects the traditional Mexican custom of social support, whereby high- and middle-income individuals help those with low or no income [19]. Impoverished people in Mexico City depend for their daily existence on those with high and middle incomes; therefore, they need to live close to high- and middle-income areas. As a result, there was no significant difference in residential location between the subjects in Group-pdm and Group-post (Table 3).

Seasonal influenza vaccination in Mexico is limited to the young and elderly [22]. Although a previous study reported that vaccination status was independently associated with H1N1 influenza [23], there was no significant difference between the groups in the present study (Table 1). Although smoking is also associated with H1N1 influenza [2], [18], there were significantly more smokers in Group-post than in Group-pdm (p = 0.002), which may reflect the fact that there were more elderly patients in Group-post (p = 0.001). In terms of comorbid conditions, more patients in Group-post had chronic respiratory illness than did those in Group-pdm (p < 0.001). These results indicate that H1N1 influenza is an emerging infectious disease that could infect individuals beyond the population without underlying respiratory illness. One year after the influenza outbreak, after some of the population had gained immunity [24], [25], the elderly population with underlying respiratory illness and who were smokers were more likely to be susceptible to influenza virus infection than the younger population without underlying respiratory illness.

The time from onset of symptoms to initiation of oseltamivir treatment is a key factor in reducing severe respiratory conditions due to H1N1 influenza [14], [15]. The time to initiation of oseltamivir treatment depended on health-care-seeking behavior. After the first manifestation of the outbreak in Mexico, the mass media drew attention to the disease and created a sense of fear in the population [18]. However, among impoverished individuals and those with less education, it may be difficult to obtain information from media sources. Although television was a major source of information for the patients in our study (Table 4), more patients in Group-post than in Group-pdm did not receive information about methods of prevention of H1N1 influenza infection and the necessity for quick access to health care (p<0.001). This indicates the importance of the method of information distribution and education for enhancing the social response to an influenza pandemic. We also evaluated the factors affecting the time from symptom onset to initiation of oseltamivir treatment (Table 5). The number of rooms in the household, receiving information about the necessity of quick access to health care, and house construction materials were evaluated as independent factors that possibly influenced health-care-seeking behavior. Poverty is associated with difficult housing conditions including the number of rooms and house construction materials. It also associated with lower access to information from media resources that could motivate people to seek early access to health care owing to a lack of utility services in the household. In addition, fewer rooms in a household was associated with increased risk of human-to-human infection. This indicates that poverty strongly influences health-care-seeking behavior and suggests the importance of distribution of information and educational resources.

The present study was limited to a population that was mostly uninsured and facing socioeconomic difficulties in Mexico City. Although there is a large gap between poverty and wealth in Mexico, the present study did not evaluate the range of socioeconomic levels in the population. Patients in Group-pdm had H1N1 influenza confirmed by RT-PCR, but the same test was not performed in patients in Group-post for budgetary reasons in the INER. Therefore, Group-post may have included patients with pneumonia not caused by influenza A(H1N1)pdm09 virus, but by some other type of influenza A virus. Further study, including an investigation of different socioeconomic populations, is needed to determine the impact of socioeconomics on the severity of disease due to influenza infection.

Although many factors affect disease occurrence and severity (including pneumonia), health-care-seeking behavior, poverty, and distribution of information are important factors from a socioeconomic point of view. These factors may explain the different patterns of morbidity and mortality for influenza A(H1N1)pdm09 in different countries and regions.
